# A Survey Exploring Inflammatory Back Pain in Patients With Inflammatory Bowel Disease

**DOI:** 10.7759/cureus.55264

**Published:** 2024-02-29

**Authors:** Onkarpreet K Jassel, Hasan Tahir, Sian Bamford, Paolo Giuffrida

**Affiliations:** 1 Internal Medicine, Royal Free London NHS Foundation Trust, London, GBR; 2 Medicine, University College London, London, GBR; 3 Physiotherapy, Royal Free London NHS Foundation Trust, London, GBR; 4 Gastroenterology, Royal Free London NHS Foundation Trust, London, GBR

**Keywords:** clinical rheumatology, diagnostic delay, axial spondyloarthritis, inflammatory bowel disease, inflammatory back pain

## Abstract

Background

Diagnostic delay of axial spondyloarthritis (axSpA) is a widely recognized issue worldwide, providing a great burden for patients with this disease. AxSpA is present in a significant proportion of patients with inflammatory bowel disease (IBD). This UK study primarily aims to identify the presence of inflammatory back pain (IBP) in patients attending IBD clinic. Further aims of this study include investigating if participants had received further referrals and diagnoses for their IBP and considering factors contributing to diagnostic delay.

Methods

Patients were recruited from a Royal Free London NHS Trust hospital’s IBD clinic. Each participant completed a 23-question survey. The Berlin criteria were applied to the questions to investigate the presence of IBP. Further questions were asked about their IBD diagnosis and treatment, the healthcare professionals they had seen for their back pain, and other extra-articular features associated with axSpA.

Results

Seventy-five patients completed the online survey sent out via email. Forty percent (n = 30) of participants were female and 60% (n = 45) were male. Sixty-one percent (n = 36) of participants from the colitis clinic reported they had back pain, and 41% of the participants reported back pain for over three months. Of these, 39% (12) of participants fulfilled the Berlin criteria for IBP. Of patients experiencing back pain for over three months, we found that 10% (3) fulfilled the Berlin criteria but had not received a diagnosis for their IBP. All patients who had fulfilled the Berlin criteria but had not received a diagnosis for their IBP had seen their general practitioner (GP) and an allied healthcare professional, but not a rheumatologist.

Conclusions

This study highlights the presence of possibly undiagnosed axSpA in patients with IBD. The reasons for the diagnostic delay of axSpA are multifactorial. We consider specific patient characteristics, lack of awareness and education of the condition, and issues in the referral process. There is a need to improve education and awareness of axSpA, reconsider referral processes, and consider new initiatives such as joint specialty clinics to identify and treat axSpA on time.

## Introduction

About 60% of the general adult population of the United Kingdom experience lower back pain in their lifetime [1]. Given this percentage, it is unsurprising that axial spondyloarthritis (axSpA) is often not initially suspected as a cause for lower back pain; however, the incidence of axSpA is around 0.5% in UK adults, more prevalent than clinicians may realize [2].

AxSpA is a chronic inflammatory disease mostly affecting the sacroiliac joints and spine but can present with extra-articular features including inflammatory bowel disease (IBD), uveitis, psoriasis, and enthesitis [3].

The burden of axSpA in patients with IBD affects up to 10% of these patients [4]. This burden is exacerbated due to the current average delay in diagnosis of axSpA being 8.5 years in the United Kingdom [5]. Longer delays can give rise to increased physical disability, which affects the quality of life of patients and also has clinical and economic implications [6,7]. Factors including the education level of patients and clinicians, sex, and being HLA-B27 negative have been shown to influence delay in diagnosis [2,3].

This study aims to investigate the presence of inflammatory back pain (IBP) in patients with IBD. It looks at how many patients with IBP have received a diagnosis of IBD and reflects on current practices to improve the identification of IBP and reduce the time to diagnose axSpA in IBD patients, aiming for the gold standard of one year to diagnosis, set by the National Axial Spondyloarthritis Charity (NASS) [2]. Identifying IBP is important for the early diagnosis of axSpA [8].

## Materials and methods

Participants with IBD were recruited from a London NHS Hospital, following their attendance at a specialist IBD clinic. Electronic surveys were sent out to patients following verbal attainment of their informed consent. Surveys were completed anonymously.

Questions about participant demographics, IBD diagnosis including the number of years since diagnosis, and IBD medications were asked in the survey. Participants were asked if they had an existing diagnosis or a family history of axSpA, how old they were when it commenced, if they had seen a healthcare professional for their back pain, and what kind of professional was seen.

They were further questioned to assess if they met the Berlin criteria, which can aid in determining the presence of IBP (Table 1) [9]. In addition to the back pain being chronic (present for more than three months), if at least two of the four Berlin criteria were met, the patient was likely to have IBP.

**Table 1:** The Berlin criteria for inflammatory back pain Source: Ref. [9].

Berlin Criteria for Inflammatory Back Pain
Morning stiffness lasting over 30 minutes
Improves with activity, but not rest
Waking in the second half of the night due to pain
Alternating buttock pain

The survey also included questions regarding the presence of enthesitis, uveitis, and psoriasis (extra-articular manifestations of axSpA). The survey that was sent out for participants to complete is included in the Appendix table.

## Results

Seventy-five participants were recruited for the survey from the IBD clinic. About 61% (n = 46) of participants reported back pain; 41% (n = 31) of participants reported that they had experienced back pain for over three months. The mean age (n = 26) of those who could recall the age at which their chronic back pain started was 35 years old. Of the 31 participants with chronic back pain, 39 (n = 12) had met the Berlin criteria for IBP. Nine of those fulfilling the criteria had been previously diagnosed with axSpA. The presence of extra-articular features in participants meeting the Berlin criteria is detailed in Table 2.

**Table 2:** Presence of extra-articular manifestations of axSpA in participants meeting the Berlin criteria for IBP axSpA: Axial spondyloarthritis; IBP: Inflammatory back pain.

Features	Percentage of Participants Experiencing the Feature
Uveitis	41.7% (n = 5)
Psoriasis	25% (n = 3)
Enthesitis	50% (n = 6)

Of those reporting back pain for over three months, three (10%) participants reported symptoms that fulfilled the Berlin criteria but had not received a diagnosis of their IBP. The ages of the undiagnosed participants ranged between 30 and 53 years old.

Two-thirds of those identified without a diagnosis were females. One of these participants had peripheral joint pain, swelling, and enthesitis, while the other participant had experienced uveitis, psoriasis, and enthesitis. Two of the participants described experiencing back pain for five and nine years, respectively.

**Figure 1:**
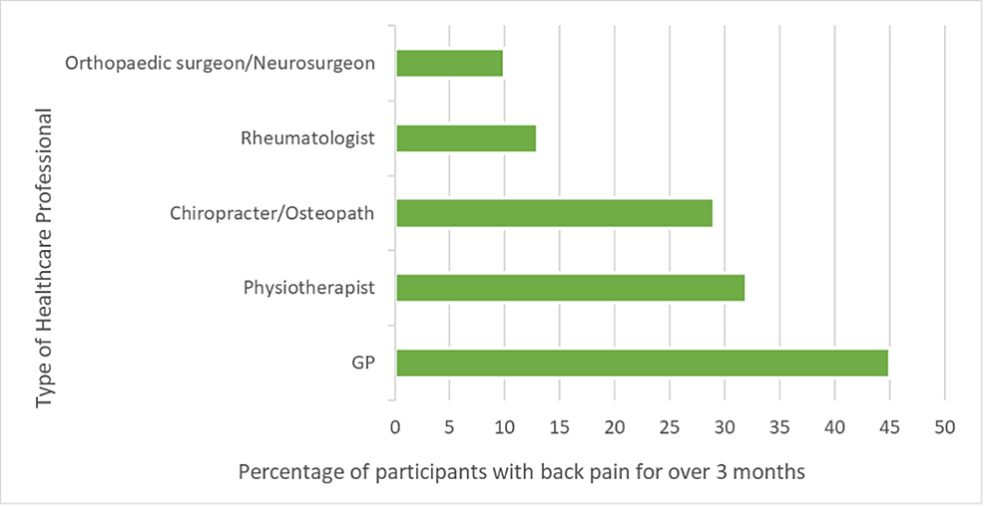
Chart showing the type of healthcare professionals visited by patients experiencing back pain for more than three months GP: General practitioner.

None of the undiagnosed patients, who had fulfilled the Berlin criteria, had seen a rheumatologist for their back pain. They had all seen their general practitioner and an allied health professional (either a physiotherapist, chiropractor, or osteopath). Figure 1 shows the percentage of patients who had sought out medical attention for their back pain from different types of healthcare professionals.

## Discussion

AxSpA affects up to 10% of patients with IBD [4]. Our study found that 41% of our cohort with IBD experienced back pain for over three months, and 16% were likely to suffer from IBP as they fulfilled the Berlin criteria. About 75% of these participants had received a prior diagnosis of axSpA.

In our cohort, we found that 25% (three out of 12) of participants with chronic back pain who fulfilled the Berlin criteria for IBP had not seen a rheumatologist and, therefore, may have undiagnosed axSpA.

The patients in our study who fulfilled the criteria but had not received a diagnosis for axSpA reported that they had their symptoms for five to nine years. A delay in diagnosis has been widely recognized; this has been averaged at 8.5 years in the United Kingdom [2,5].

A young age of onset has been recognized by multiple healthcare professionals (primary and secondary care doctors, chiropractors, and osteopaths) as a potential barrier to diagnosis [3,10,11]. Our findings of a mean age of symptom onset of 35 years are in keeping with these studies. This could be due to the lack of continuity of care, with younger patients presenting to urgent care centers, receiving pain relief, and being discharged without further investigation. Additionally, axSpA has an insidious onset, so signs and symptoms may take some time to develop to be identified by clinicians.

It is important to note the reluctance of patients to seek a medical opinion and accept a larger diagnosis as the young age of onset coincides with an important time personally for patients when considering careers and personal relationships [3].

Another patient factor that may influence the delay in diagnosis could be attributed to the female sex; we found that two-thirds of our undiagnosed IBP patients were females. This inflammatory condition has been viewed in the past as a male disease, which may skew a clinician’s differentials [12].

Back pain is a common presentation in the adult population, with 61% of participants reporting all types of back pain. Education of healthcare professionals is required to correctly differentiate axSpA from more common types of back pain. We found that all patients identified as having a possible undiagnosed axSpA had not seen a rheumatologist but had seen their GP and either a physiotherapist or osteopath/chiropractor.

A lack of collaboration between healthcare professionals has been expressed by Yong et al. with chiropractors and osteopaths expressing non-confidence in commencing a referral process for suspected IBP [3]. Patients without onward referral from both primary and secondary care services have been identified as playing a role in the delayed diagnosis of axSpA [10,13,14].

The need for increased education of healthcare practitioners regarding axSpA is evident in the literature. A study conducted by van Onna et al. demonstrated that after receiving education on axSpA, there was a significant increase in GPs correctly identifying axSpA [13].

Additionally, further education is required in secondary care. A survey distributed in the United Kingdom identified that IBD nurses have not received education on axSpA, and while consultant gastroenterologists had received education on this matter, less than two-thirds felt confident in recognizing the inflammatory condition. Relatively low-cost and straightforward measures such as posters in gastroenterology clinics, which simply show the signs of axSpA and guidance for referral, accessible to both patients and clinicians are suggested [14].

A Canadian study piloted the Toronto axial spondyloarthritis questionnaire in inflammatory bowel disease (TASQ-IBD) to address the delayed diagnosis of axSpA in at-risk patients. Adopting a case-finding questionnaire like this for patients with IBD and back pain or stiffness could be a valuable tool in facilitating timely diagnoses [15].

Joint rheumatology and gastroenterology clinics could aid in the timely diagnosis of concurrent axSpA and IBD. This is an initiative that has been trialed in Italy with positive results in identifying axSpA in IBD patients [16].

Limitations

As the study conducted involved a survey, we consider the presence of recall bias from participants. We also acknowledge that participants were recruited from only one NHS center and that the sample size was limited.

## Conclusions

We have shown that patients with IBD are potentially living with and suffering from the undiagnosed burden that axSpA brings. Three out of 31 (10%) participants with back pain potentially have undiagnosed axSpA. Diagnostic delay is multifactorial; we have considered patient characteristics, issues in the referral process, and lack of awareness. This study highlights the importance of educating healthcare professionals and patients about this condition to promptly diagnose, treat, and ultimately improve the quality of life of axSpA patients. A place for joint clinics with rheumatologists and gastroenterologists has been identified to assist in the identification and onward referral of patients with possible axSpA.

## Appendices

**Table 3:** A 23-question survey that was sent to the participants D.O.B.: Date of birth; NSAIDs: Non-steroidal anti-inflammatory drugs; GP: General practitioner.

	Survey Questions					
1	Have you been diagnosed with inflammatory back pain/ankylosing spondylitis/axial spondyloarthritis?					
	Yes	No				
2	D.O.B?					
3	Gender					
	Male	Female	Other	Prefer not to say		
4	What is your inflammatory bowel disease diagnosis?					
	Crohn's disease	Ulcerative colitis	Indeterminate colitis			
5	How long (in years) have you had inflammatory bowel disease?					
6	Are you currently taking biologic medication?					
	Yes	No				
7	If yes, which biologic are you currently taking?					
	Infliximab (Remicade, Remsima, Inflectra, Flixabi, Zessly)	Adalimumab (Humira, Amgevita, Hulio, Imraldi, Hyrimoz)	Vedolizumab (Entyvio)	Ustekinumab (Stelara)	Tofacitinib (Xeljanz)	Golimumab (Simponi)
8	If no, have you ever taken biologic medication? (Select all relevant)					
	Infliximab (Remicade, Remsima, Inflectra, Flixabi, Zessly)	Adalimumab (Humira, Amgevita, Hulio, Imraldi, Hyrimoz)	Vedolizumab (Entyvio)	Ustekinumab (Stelara)	Tofacitinib (Xeljanz)	Golimumab (Simponi)
9	Do you experience back pain that has lasted for longer than 3 months?					
	Yes	No				
10	If yes, does it improve with NSAIDs, i.e., ibuprofen, naproxen?					
	Yes	No				
11	How old were you (in years) when your back pain started?					
12	Where is your pain located? (Select all relevant)					
	Neck	Mid back	Lower back	N/A		
13	Does your back pain improve with activity or rest?					
	Activity	Rest	Neither	N/A		
14	Have you ever seen a healthcare professional regarding your lower back pain?					
	Yes	No				
15	If yes, which healthcare professionals have you seen? (Select all relevant)					
	GP	Physiotherapist	Chiropractor/Osteopath	Orthopedic/Neurosurgeon	Rheumatologist	
16	Do you experience morning stiffness lasting longer than 30 minutes?					
	Yes	No				
17	Do you wake during the second half of the night due to back pain?					
	Yes	No				
18	Do you have alternating buttock pain?					
	Yes	No				
19	Do you experience pain and swelling in other joints?					
	Yes	No				
20	Have you ever had plantar fasciitis (pain in the heel/arch of your foot) or Achilles tendinitis (pain in the tendon at the back of your ankle)?					
	Yes	No				
21	Have you ever had uveitis/eye inflammation/a red eye?					
	Yes	No				
22	Have you ever had psoriasis?					
	Yes	No				
23	Does anyone in your family have ankylosing spondylitis					
	Yes	No				
